# Determination of the differentiation capacities of murines' primary mononucleated cells and MC3T3-E1 cells

**DOI:** 10.1186/1475-2867-10-42

**Published:** 2010-10-28

**Authors:** Muhammad Dain Yazid, Shahrul Hisham Zainal Ariffin, Sahidan Senafi, Mohamad Abdul Razak, Rohaya Megat Abdul Wahab

**Affiliations:** 1 School of Biosciences and Biotechnology, Faculty of Science and Technology, Universiti Kebangsaan Malaysia, 43600 Bangi, Selangor, Malaysia; 2 Department of Orthodontics, Faculty of Dentistry, Universiti Kebangsaan Malaysia, 50300 Kuala Lumpur, Malaysia; 3 Department of Orthopedic, Universiti Kebangsaan Malaysia Medical Centre, Universiti Kebangsaan Malaysia, 56000 Cheras, Selangor, Malaysia

## Abstract

**Background:**

The main morphological features of primitive cells, such as stem and progenitor cells, are that these cells consists only one nucleus. The main purpose of this study was to determine the differentiation capacities of stem and progenitor cells. This study was performed using mononucleated cells originated from murine peripheral blood and MC3T3-E1 cells. Three approaches were used to determine their differentiation capacities: 1) Biochemical assays, 2) Gene expression analysis, and 3) Morphological observations.

**Results:**

We found that both cells were able to differentiate into mature osteoblasts, as assayed by ALP activity. RT-PCR analysis showed the activation of the *Opn *gene after osteoblast differentiation. Morphological observations of both cells revealed the formation of black or dark-brown nodules after von Kossa staining. Nevertheless, only mononucleated cells showed the significant increase in TRAP activity characteristic of mature osteoclasts. The osteoclast-specific *CatK *gene was only upregulated in mononucleated cells. Morphological observations indicated the existence of multinucleated osteoclasts. *Sca-1 *was activated only in undifferentiated mononucleated cells, indicating that the cells were hematopoietic stem cells. In both cell lines, the housekeeping *Gapdh *gene was activated before and after differentiation.

**Conclusion:**

The isolated mononucleated cells were able to differentiate into both osteoblasts and osteoclasts; indicating that they are stem cells. On the other hand, MC3T3-E1 cells can only differentiate into osteoblasts; a characteristic of progenitor cells.

## Background

The advent of stem cell technology provides remarkable opportunities for the improvement and extension of human life. Stem cell research has spread into many fields of study, indicating that it represents an area with great scientific and therapeutic promise. Their unique ability to self-renew indefinitely and to differentiate into multiple cell types can make them useful to elucidate normal cellular processes as well as to understand their mechanisms [1]. In contrast, progenitor cells are unipotent proliferative cells with a limited capacity for self-renewal. The unipotency of progenitor cells is depending on the type of their parent stem cell as well as their physiological niche. Progenitors are said to be in a more advanced stage of cell differentiation [2] that is between multipotent stem cells and fully differentiated cells.

Opinion among researchers regarding the definitions of stem and progenitor cells is still evolving. For example Seaberg and van der Kooy [3] had stated that some researchers exclude the characteristic of stem cells plasticity as one of the novel property of stem cells. They also suggested that there are differences in the biological marker of these two cell types, i.e., hematopoietic stem cells and progenitor cells detected in both *in vivo *and *in vitro *studies. Therefore, a few potential biological markers, such as *Thy-1 *and *Sca-1 *have been used to prove the difference for respective cells.

However, the low expression of *Thy-1 *makes *Sca-1 *as a chosen hematopoietic stem cell marker. *Sca-1 *is a membrane-anchored protein from the murine Ly-6 family mouse strain. *Sca-1 *regulates hematopoietic stem cell self-renewal and the development of specific progenitor populations, such as blood cells [4]. Its expression has been extensively used as a marker of hematopoietic stem cells, [4,5] thus there is no expression of *Sca-1 *in progenitor cells.

Cellular differentiation has been extensively studied for many years and has become one of the most important areas of research, especially in the medical field. Information about the cells development gained from biochemical assays, characterizations of cell morphology, and gene expression is being used to elucidate the fundamental mechanisms of cell differentiation. The differentiation of stem and progenitor cells into specialized cell types in charge of bone remodelling is important for the maintenance of mineral homeostasis and the repair of microfractures. Skeleton growth and bone remodelling are continual processes involving two specialized cells known as osteoblasts, which deposit the organic and inorganic components of the bone matrix, and osteoclasts, which remove bone matrix [6,7]. The research of Duplomb et al [8] on osteoblast and osteoclast differentiation using embryonic stem cells has contributed to the understanding and harnessing of bone homeostasis.

The maintenance of bone homeostasis is essential for the functional skeleton, including skeletal growth, repair of skeletal damage and replacement of aged bone. Bone remodelling is a continual process, which involves osteoblast and osteoclast cells, which are originate from different cell lineage [9]. Osteoblasts are believed to originate from common mesenchymal progenitors, known as multipotent mesenchymal stem cells [10]. These multipotent mesenchymal stem cells have been studied extensively for their capability to give rise to a number of cell lineages, such as adipocytes, myoblasts and chondrocytes. Importantly, the differentiated osteoblast is responsible for the formation of new bone matrix. In their *in vivo *microenvironment, osteoblasts also produce factors that regulate the formation of osteoclasts and osteoclastic activity [7]. Osteoclasts are multinucleated cells derived from hematopoietic stem cells and are responsible for bone resorption. Bone resorption is important in many skeletal processes, e.g., bone growth, tooth eruption and fracture healing [11]. Both osteoblasts and osteoclasts originate from different lineage, therefore it can be used to prove the capacity of stem cell to differentiate into other cell lineage rather than progenitor cells.

During the process of differentiation and/or maturation, the expression patterns of many genes are modulated in pluripotent cells. The specific expression pattern of a collection of known genes during this process has established them as differentiation markers [12]. Osteoblasts express several genes as markers of their differentiation, including osteopontin, alkaline phosphatase and osteocalcin [7,13-16]. However, osteopontin is one of the potential markers, which was widely used among researchers in osteoblasts study along with alkaline phosphatase [16-18].

Osteopontin (*Opn*), also known as early T cell activation gene-1, or *eta-1*, is a secreted, acidic glycoprotein with pleiotropic effects. The interaction of *Opn *with integrins and CD44 activates several different signaling pathways during the differentiation process [17]. Beck et al [16] used gene array technology to explain changes in gene expression during the differentiation of the mouse calvarial-derived MC3T3-E1 cell line and osteopontin was one of the genes identified as a biological marker. Kumar et al [18] used osteopontin promoters to transduce mouse mesenchymal stem cells before evaluating the expression of this bone-specific promoter.

Osteoclasts were shown to synthesize and secrete cathepsin K (*CatK*) into the extracellular compartment at the attachment site between osteoclasts and the bone surface [19]. Recent investigations have shown that *CatK*, a lysosomal enzyme is synthesized specifically by cells in the osteoclast lineage [20,21]. Therefore, *CatK *is an essential marker of bone resorption.

Morphological characterizations of osteoblasts and osteoclasts are evaluated by von Kossa and May Grunwald-Giemsa staining, respectively. For osteoblasts, alkaline phosphatase expression is considered an early differentiation marker, while von Kossa staining identifies the late differentiation stage of osteoblasts due to its capability to label calcium minerals [11]. The mineralized nodule formation in mature osteoblasts can be revealed by von Kossa silver staining and visualized as metallic silver. Similar study by Tsuang et al [11] using this staining have verified the mineralization nodules formation of osteoblastic cell that been affected by caffeine. On the other hand, May Grunwald-Giemsa staining is used to observe the nuclear morphology of osteoclasts. The nucleus is stained by May Grunwald, while Giemsa stains the cytoplasm, thus highlighting the typical characteristics of mature multinucleated osteoclast cells. May Grunwald-Giemsa staining were widely used in cellular and microbiological research. Recent studies using May Grunwald-Giemsa staining on Flt3+ bone marrow progenitor cells able to show the existence of osteoclastic differentiated cells in the presence of M-CSF and RANKL [22,23]. In this study, we applied von Kossa and May Grunwald-Giemsa staining on both primary mononucleated and MC3T3-E1 cells to observe the ability of these to differentiate into osteoblast and osteoclast cells respectively.

Therefore, we aimed to characterize the differences between stem and progenitor cells *in vitro *by analyzing the differentiation capacities of these cells using three approaches: biochemical assays, morphological characterization and genetic analysis involving *Opn*, *CatK *and *Sca-1 *expressions. The TRAP and ALP assays permit the simultaneous analysis of both of the bone metabolism enzymes tartrate-resistant acid phosphatase (TRAP) and alkaline phosphatase (ALP). Morphological observations were based on the formation of calcium nodules, as determined by von Kossa staining, and large multinucleated cells, as determined by May Grunwald-Giemsa staining, in osteoblasts and osteoclasts, respectively. Finally, the activation of the *Opn *gene in osteoblasts and *CatK *in osteoclasts supported the identification of each cell type.

## Results

### Biochemical Assays

Two different biochemical assays were carried out to measure osteoblast and osteoclast differentiations: alkaline phosphatase (ALP) and tartrate resistant acid phosphatase (TRAP) assays. ALP is a biochemical marker of osteoblasts, while TRAP is a biochemical marker of osteoclasts, as the ALP and TRAP enzymes are secreted during osteoblast and osteoclast differentiation, respectively.

**Figure 1:**
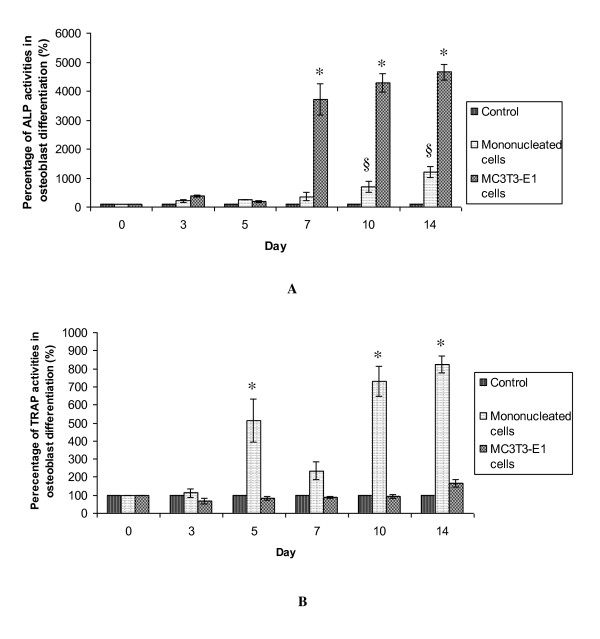
**ALP and TRAP activities in osteoblast differentiation**. (A) ALP activities during osteoblast differentiation. There was significantly increased ALP activity in mononucleated cells at days 10 and 14 of differentiation, while MC3T3-E1 cells showed significant increases at days 7, 10 and 14 of differentiation. (B) TRAP activities during osteoblast differentiation. There were significant increases of TRAP activity in mononucleated cells at days 5, 10 and 14 of osteoblast differentiation. The TRAP activity in MC3T3-E1 remains constant and similar to control activity. Results are presented as mean ± SD (n = 3). The * and § indicate significant difference (p < 0.05) of mononucleated cells and MC3T3-E1 respectively as compared with day 0.

Figure 1A shows the percentage of ALP activity during osteoblast differentiation. ALP activity in mononucleated and MC3T3-E1 cells gradually increased from day 3 to day 14 of osteoblast differentiation. This increment was statistically significant (p < 0.05) at days 7, 10 and 14 for MC3T3-E1 cells, and this was true at days 10 and 14 for mononucleated cells. Therefore, both cell types successfully differentiated to osteoblasts. As depicted in Figure 1B, TRAP activity in mononucleated cells during osteoblast differentiation fluctuated from the beginning of differentiation. The activity was significantly increased at day 5, decreased at day 7 and significantly increased at days 10 and 14. TRAP activity in MC3T3-E1 cells remained constant similar to control. The control is where the cells were not exposed to the differentiation factors.

**Figure 2:**
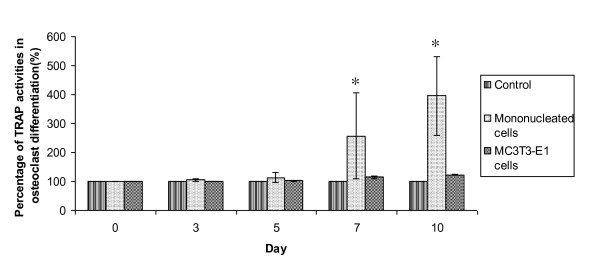
**TRAP assay in osteoclast differentiation**. Assays were performed after 3, 5, 7 and 10 days of differentiation. The graph shows that only mononucleated cells exhibited a significant increase of TRAP activity after 7 and 10 days of osteoclast differentiation. TRAP activity in MC3T3-E1 remained constant, similar to control activity. Results are presented as mean ± SD (n = 3). The * indicate significant difference (p < 0.05) of mononucleated cells and MC3T3-E1 respectively as compared with day 0 activity for the same culture period.

In osteoclast differentiation, the ALP activity of mononucleated cells was unstable and not significantly different (p > 0.05) from the control. In addition, the ALP activity in MC3T3-E1 cells also remained similar to the control activity. During the TRAP assay, only mononucleated cells showed an increase of TRAP activity starting from day 3 of osteoclast differentiation induction. As shown in Figure 2, there was a significant increase of TRAP activity at days 7 and 10 of osteoclast differentiation, indicating that the mononucleated cells differentiated into osteoclasts. In contrast, MC3T3-E1 cells showed similar TRAP activities to the controls (Figure 2). Therefore, enzymological assays demonstrated that successful osteoblast and osteoclast differentiation only occurred in mononucleated cells.

### Activation of *Opn, CatK, Sca-1 *and *Gapdh *Genes

We compared the gene expression profiles of mononucleated and MC3T3-E1 cells in the undifferentiated and differentiated states to further confirm their differentiation capacities. Previous studies have shown that there are several specific gene markers for osteoblasts, for example, osteopontin (*Opn*), alkaline phosphatase (*Alp*) and osteocalcin (*Ocn*) [7,13,15,16]. The gene markers we used for osteoclasts were tartrate-resistant acid phosphatase (*Trap*) and cathepsin K (*CatK*) [9].

**Figure 3:**
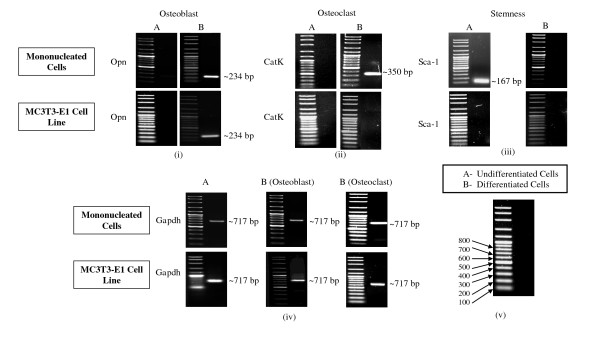
**Expression of specific gene markers**. The RT-PCR analysis was performed using RNA isolated from mononucleated and MC3T3-E1 cells before and after differentiation into osteoblast and osteoclast cells. (i) The expression of *Opn *(234 bp) indicates differentiation into mature osteoblasts. (ii) *CatK *(350 bp) expression shows osteoclast differentiation. (iii) The activation of *Sca-1 *(167 bp) is indicative of the degree of stemness of the cells. (iv) *Gapdh *(717 bp) was used as a positive control. (v) The labelled 100 bp DNA marker to identify the approximate size of amplicons. Equal volumes of the products were separated in agarose gels using 100 bp DNA markers and stained with ethidium bromide.

The RT-PCR analysis showed the activation of *Opn *expression in mononucleated and MC3T3-E1 cells after being cultured for 14 days in osteoblast differentiation media. The amplification of the *Opn *transcript, with a size of 234 bp, is shown in Figure 3i. For the osteoclast differentiated cells, the expression of *CatK *was detected only in mononucleated cells after 10 days of culturing in osteoclast differentiation media. As shown in Figure 3ii, only the mononucleated cells contained the 350-bp *CatK *transcript.

The *Sca-1 *gene was activated only in undifferentiated mononucleated cells, as shown by the amplification of the 167-bp *Sca-1 *transcript (Figure 3(iii)). The *Gapdh *gene was activated in both cell lines, before and after differentiation, as shown by the RT-PCR product of 717 bp (Figure 3iv). All the amplified cDNA, i.e. *Opn, CatK, Sca-1 *and *Gapdh*, were subjected to DNA sequencing and verified using blastn program from NCBI.

### Morphological Analysis of Differentiated Cells

**Figure 4:**
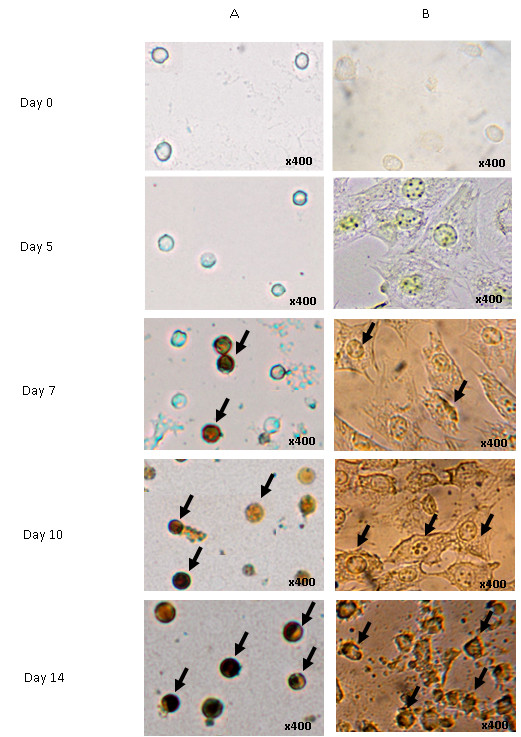
**Morphology of differentiated osteoblast cells**. (A) Morphology of differentiated murine mononucleated cells. (B) Morphology of differentiated MC3T3-E1 cells. Both cells were cultured in osteoblast differentiation media for 5, 7, 10 or 14 days prior to morphological analysis by von Kossa staining. The black arrows show the formation of light-brown, dark-brown or black nodules (400× magnification).

Figure 4A and 4B show the differentiated osteoblasts from mononucleated and MC3T3-E1 cells, respectively. Both cells were able to differentiate into osteoblasts. We visualized the extent of their differentiation into osteoblasts with von Kossa staining, which produces stained calcium nodules. The black arrows show the formation of nodules in differentiated mononucleated cells (Figure 4A) and MC3T3-E1 cells (Figure 4B).

**Figure 5:**
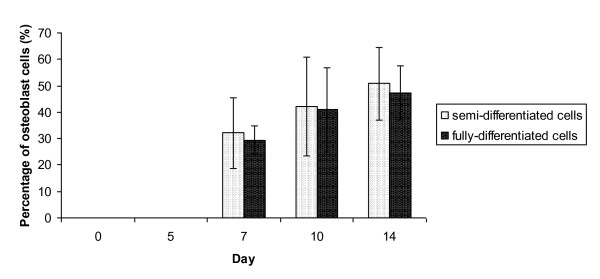
**Percentage of cells differentiated into osteoblast**. The percentages of differentiated cells were calculated upon 14 days of osteoblast differentiation and classified into two categories, semi-differentiated and fully differentiated osteoblasts.

We quantified the percentage of differentiated osteoblasts from mononucleated cells and found that they could be classified into three distinct classes, according to the color of the von Kossa-stained nodules: undifferentiated, semi-differentiated and fully-differentiated cells. The undifferentiated cells (controls) did not show any stained nodules. A light-brown color represented the semi-differentiated cells, while dark-brown or black cells were fully differentiated cells. Figure 5 shows that at day 7, ~32% of the mononucleated cells were semi-differentiated into osteoblasts, while ~29% of the mononucleated cells were fully differentiated osteoblasts. At day 10 of differentiation, the percentages were increased to 42% and 40%, respectively. At the end of osteoblast differentiation (day 14), the percentage of semi-differentiated osteoblasts increased to 50%, while fully differentiated osteoblasts made up 48%. These results show the percentages of semi-differentiated and fully-differentiated osteoblasts were increased upon differentiation. Although semi-differentiated and fully differentiated osteoblasts were increased gradually, the percentage of fully differentiated osteoblast cells was less than semi-differentiated osteoblasts (Figure 5). After 14 days differentiation, we found that the cells were still not 100% differentiated. Other studies have demonstrated that osteoblast were 100% differentiated until 21 days cultured in osteoblast differentiation media [24]. Nevertheless, our study was only to indicate that both the primary mononucleated and MC3T3-E1 cells have capability to differentiate into osteoblast.

**Figure 6:**
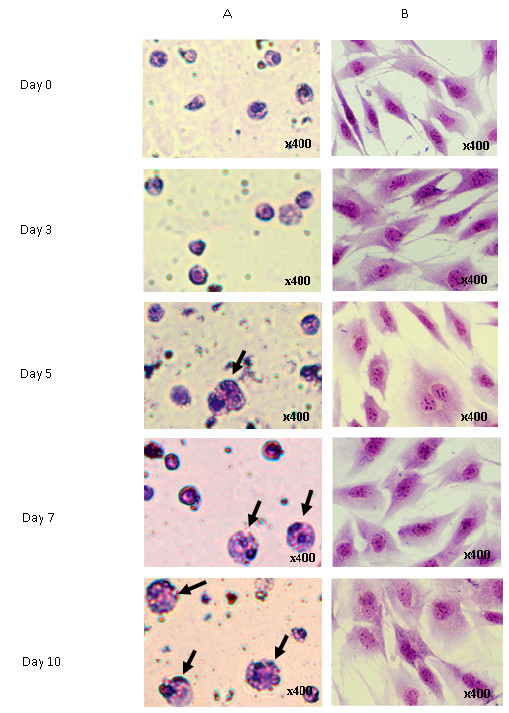
**Morphology of differentiated osteoclast cells**. May Grunwald-Giemsa staining of (A) multinucleated osteoclasts and (B) MC3T3-E1 cells. Both cells were subjected to cytospin at day 3, 5, 7 or 10 differentiations prior to May Grunwald-Giemsa staining. The figure shows that the numbers of multinucleated osteoclast cells increased upon differentiation of mononucleated cells but not MC3T3-E1 cells. The black arrows show the multinucleated osteoclasts (400× magnification).

Mature osteoclasts have more than one nucleus and are very large in size [11,25]. The nucleus is stained as dark purple with May-Grunwald Giemsa, while the cytoplasm is stained lighter (pink rose) than the nucleus. No multinucleated cells formed in the undifferentiated cells (control). Mononucleated cells cultured in osteoclast differentiation media for 10 days exhibited the aforementioned criteria of osteoclast cells, as shown by the black arrows (Figure 6A). In contrast, MC3T3-E1 cell morphology did not show any multinucleated cells or size changes even after 10 days in osteoclast differentiation media. Therefore, the MC3T3-E1 cells did not differentiate into osteoclasts (Figure 6B). These data indicate that only mononucleated cells had the capacity to differentiate into osteoclasts.

**Figure 7:**
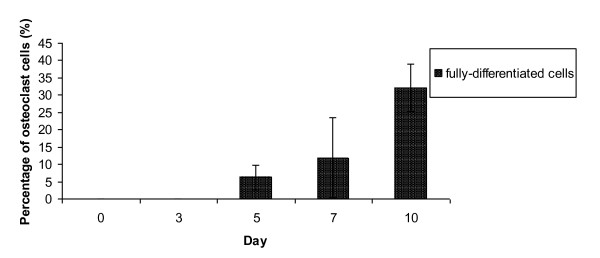
**Percentage of cells differentiated into osteoclast**. The percentages of differentiated cells were calculated upon 10 days of osteoclast differentiation.

From these data, we were able to quantify the percentage of osteoclast-differentiated cells according to two different classes, undifferentiated and differentiated cells. Photographs of the cells were selected at random and counted prior to the percentage analysis. The percentage of fully differentiated osteoclasts increased with time (Figure 7). Approximately 6% fully differentiated osteoclasts were observed at day 5 of osteoclast differentiation. The percentage of fully differentiated osteoclast cells increased to 12% and 32% at days 7 and 10 of osteoclast differentiation, respectively (Figure 7).

## Discussion

The differentiation of stem cells is widely studied because of their biological properties and potential medical importance. Unfortunately, the ambiguity of the distinctions between stem and progenitor cells continues to puzzle stem cell researchers. Therefore, we studied the differentiation of stem cells primarily isolated from murine peripheral blood and progenitor cells originating from mouse calvaria to compare their differentiation capacities. The approaches that we took showed that the mononucleated cells were able to differentiate into both osteoblasts and osteoclasts, whilst the MC3T3-E1 cells could only differentiate into osteoblasts. Therefore, this study demonstrates that the primary mononucleated cells are stem cells, but the osteoblast progenitor MC3T3-E1 cell line is not.

ALP enzyme activities at day 14 were highest when mononucleated and MC3T3-E1 cells were cultured in osteoblast differentiation media. These data proved that both cells were differentiated into osteoblast cells. The increment of ALP activity during osteoblasts differentiation was also proved by Wang et al [24] and Zhao et al [25]. Their studies shows that MC3T3-E1 pre-osteoblast was differentiated into osteoblast cells after induced with ascorbic acid and β-glycerophosphate for 14 days.

The complement of the osteoblast is the osteoclast, which is multinucleated and large in size. A large amount of TRAP enzyme is secreted during osteoclast differentiation. TRAP was secreted at the highest levels at day 10 of osteoclast differentiation (Figure 2). We performed a TRAP assay to detect the degree of osteoclast differentiation in both cell lines and found that TRAP activity was significantly increased only in mononucleated primary cells. These data are in accord with the findings of Intan Zarina et al [13,14], who reported that TRAP activity is increased at day 10 of osteoclast differentiation. The mononucleated cells, therefore, had the capacity to differentiate into both osteoclasts and osteoblasts.

Nevertheless, there were fluctuations of TRAP activity in the mononucleated cells during osteoblast differentiation. This result was similar to that of Perez-Amodio et al [26], who found fluctuating TRAP activity during the first 7 days of osteoblast differentiation. They proposed that the fluctuation of TRAP activity may have been caused by the activation and deactivation of TRAP by cysteine proteinases [27]. Therefore, the fluctuation of TRAP in our study may have had a similar cause.

In contrast, the MC3T3-E1 cells were intrinsically programmed to differentiate into their committed target cells, as they were unable to differentiate into osteoclasts. MC3T3-E1 is known to be an osteoblast progenitor committed to a specific lineage, which originated from mesenchymal stem cells [28]. When the MC3T3-E1 cells were cultured in osteoclast differentiation medium, the ALP and TRAP activities remained constant, similar to the control cells. These results indicate that MC3T3-E1 cells lack the potential to differentiate into cells other than osteoblasts.

The activation of genetic markers for each differentiated cell type is a reliable indicator for the successful differentiation of cells *in vitro*. The activation of *Opn *demonstrates that the cells have differentiated into osteoblasts, while the activation of *CatK *indicates osteoclast differentiation. In this study, we showed that *Opn *and *CatK *genes were activated in the primary mononucleated cells that had been differentiated into osteoblasts and osteoclasts, respectively.

Based on rat calvarial osteoblast-like (ROB) cell studies, *Opn *is expressed during day 13 to day 15 of osteoblast differentiation. ROB cells can be fully differentiated into matured osteoblasts [28]. Other studies have reported that *Opn *expression is modulated by osteoblast stimulation *in vivo*, resulting in dramatically increased *Opn *[17,29]. We showed that both primary mononucleated and MC3T3-E1 cell lines were able to be differentiated into matured osteoblast cells.

Differentiation of mononucleated cells into mature osteoclasts was indicated by the activation of the *CatK *gene. The *CatK *gene has been widely used as an osteoclast marker in many previous studies. Hu et al [30] reported that the expression of *CatK *is increased during osteoclast differentiation in myeloid progenitor cells. Another recent study showed increased *CatK *expression during osteoclastogenesis [31]. The function of *CatK *in osteoclasts could account for their osteoclastic activities. According to Väänänen et al [32], a high level of *CatK *expression secreted by osteoclasts in the lacuna is an indicator of the resorption process. Another study showed that *CatK *is expressed during the resorption of calcium deposits in calcified tendonitis of the rotator cuff tendons, suggesting *CatK *as a potential osteoclast marker [20]. Our study shows that the activation of *CatK *was found only in mononucleated cells after 10 days cultured in osteoclast differentiation media. This is an indication that only mononucleated cells were able to differentiate into osteoclast, but not the MC3T3-E1 cell line.

Cells express many genes responsible for cell survival, such as *Gapdh *(glyceraldehyde-3-phosphate dehydrogenase) and *β-actin*, which are involved in glycolysis and cell development, respectively [33]. These housekeeping genes are frequently used to normalize mRNA levels between different samples. However, the expression of these genes may vary among tissues or cells and may change under certain circumstances [34]. In this study, *Gapdh *was used as a positive control for primary mononucleated and MC3T3-E1 cells. Its expression was constant in both types of cells, before and after differentiation. The RT-PCR amplification product of *Gapdh *(717 bp) remained activated in all respective cells and conditions (Figure 3), indicating that the cells undergo survival processes [35].

As both types of cells are derived from mice, the cells were examined for *Sca-1 *(stem cell antigen-1) activation. We found that only undifferentiated mononucleated cells activate the *Sca-1 *gene (Figure 3). *Sca-1 *is encoded by the strain-specific *Ly-6 E/A *gene, and its expression revealed that mononucleated cells are hematopoietic stem cells [4].

The morphological analysis in this study revealed that both cell types became brown or dark-brown from day 7 until day 14 of osteoblast differentiation (Figure 4). When the cells are treated with a silver nitrate solution, the silver is deposited by replacing the calcium that had been reduced by the strong light, thereby allowing the mineralized calcium nodules to be visualized as metallic silver. After von Kossa staining, osteoblasts can be visualized as brown or black, representing nodules that contain calcium salts. Previous studies have reported that mesenchymal stem cells differentiated into chondrogenic and osteogenic cells deposit calcium mineral nodules revealed by von Kossa staining [36]. The presence of calcium nodules concomitant with the increase of ALP activity and the activation of the *Opn *gene showed that the respective cells had differentiated into matured osteoblasts.

In some cases, the cells became light-brown, indicating they were less developed (Figure 4A). Tsuang et al [11] used von Kossa staining to quantify the formation of mineralization nodules in osteoblasts cultures. This is most notable when comparing semi-differentiated and fully differentiated osteoblasts. We categorized the semi-differentiated and fully differentiated osteoblasts based on the color intensity of the stained nodules. Light-brown color represented the semi-differentiated cells, while dark-brown or black indicated fully differentiated cells. Our observations showed that mononucleated and MC3T3-E1 cells had a slightly higher proportion of semi-differentiated cells than fully differentiated osteoblasts (Figure 5).

On the other hand, the significant increment of TRAP activity and the activation of *CatK *during osteoclast differentiation reflected the increased number of fully differentiated osteoclasts. The multinucleated cells, which are quite large in size, were observed at least after 5 days culturing in osteoclast differentiation media. From the quantification percentage of both differentiated cells, we found that not all cells were differentiated into osteoblasts and osteoclasts (Figure 5 and 7). We believe that this could have resulted from differences in the stemness of the cells and the heterogeneity of our isolated cells. In the *in vitro *system, not all of the heterogeneous cells differentiated when induced with osteoclastic medium [37]. This phenomenon was studied by Stockholm et al [38], who suggested that the differentiation of heterogeneous cell populations, especially stem cells, depends on extrinsic and intrinsic factors. These extrinsic and intrinsic factors may lead to changes in the gene expression patterns among heterogeneous cells.

## Conclusion

In conclusion, only mononucleated cells successfully differentiated into both osteoblasts and osteoclasts *in vitro*, whereas MC3T3-E1 only differentiated into osteoblasts. The differentiation capacities of these stem and progenitor cells have been determined and characterized, suggesting that the capacity to differentiate into more than one type of cell is only present in the (mononucleated) stem cells. Our study involved cells primarily isolated from peripheral blood. Nonetheless, these distinctions between the cell types should be emphasized rather than ignored, as they can be used to test specific hypotheses in the controversial area of stem cell definition and distinction.

## Methods

### Primary Cell Isolation and MC3T3-E1 Cell Line Culture

Peripheral blood mononucleated cells from buffy coats obtained from healthy 4-to 6-week-old mice were isolated by density centrifugation with Ficoll Paque™ Plus (G.E Healthcare, UK). Briefly, the buffy coat preparations were diluted 1:3 with Hanks' Balanced Salt Solution (HBSS) (Sigma, USA). Diluted buffy coat was laid onto 1.5 mL of Ficoll Paque™ Plus and centrifuged at 400 × g for 30 min at room temperature. The buffy coat was separated into four layers of different densities, consisting of granulocytes and erythrocytes at the bottom, the polymorphonuclear cells in the Ficoll Paque layer, mononuclear cells above that, and plasma at the top. The peripheral blood mononucleated cells were recovered after discarding the plasma, washed twice with phosphate-buffered saline (PBS) (Sigma, USA), and cultured in selective proliferation media consisting of α-Minimal Essential Medium (α-MEM) (Invitrogen, USA) supplemented with 10% (v/v) newborn calf serum (NBCS) (Invitrogen, USA) and penicillin-streptomycin solution (with final concentration of 500 unit/mL penicillin G sodium and 500 μg/mL streptomycin sulfate) (Invitrogen, USA) for 14 d prior to analysis of osteoblast and osteoclast differentiation. The selective proliferation media was then supplemented with differentiation factors, including 50 μg/mL ascorbic acid (Sigma, USA) and 10 mM β-glycerophosphate (Sigma, USA), to differentiate mononucleated cells into osteoblast cells. For osteoclast differentiation, 10 ng/mL RANKL (Peprotech, USA) and 5 ng/mL M-CSF (Peprotech, USA) were added to the proliferation medium.

Progenitor cells of the MC3T3-E1 subclone C14 (MC3T3-E1/C14) pre-osteoblast cell line (ATCC No: CRL-2596™) were used in this study. Cells were maintained in α-MEM supplemented with 1 mM sodium pyruvate (Sigma, USA) plus 10% (v/v) fetal bovine serum (FBS) (Invitrogen, USA). For differentiation assays, cells were plated at 5 × 10^4^cells/mL. As mentioned previously, differentiation was induced by the addition of 50 ng/mL ascorbic acid (Sigma, USA) and 10 mM β-glycerophosphate (Sigma, USA) to standard growth medium for osteoblasts differentiation. For osteoclast differentiation, 10 ng/mL RANKL (Peprotech, USA) and 5 ng/mL M-CSF (Peprotech, USA) were added to the proliferation medium. The medium was changed every 3-4 d.

### Alkaline Phosphatase (ALP) Assay

The cells were washed with complete growth medium followed by PBS. The whole cells were incubated in 0.1 M NaHCO_3_-NaCO_3 _buffer (pH 10), containing 0.1% (v/v) Triton X-100, 2 mM MgSO_4 _and 6 mM p-nitrophenol phosphate (pNPP) for 30 min at 37°C. The reaction was stopped by adding 1 mL 1.5 M NaOH, and the absorbance was measured at 405 nm. The ALP activity is represented as specific activity. The specific activity was determined by unit activity per total protein content (mg) and protein content was measured using Bradford method. One unit of ALP activity represents the hydrolysis of 1 μM pNPP per minute at 37°C. The ALP specific activity was presented in percentage value, which was compared to ALP specific activity in control cells (cells culture in standard growth medium) that act as basal activity (100%).

### Tartrate-Resistant Acid Phosphatase (TRAP) Assay

The cells were washed with PBS and incubated with 50 μL sodium acetate buffer (50 mM, pH 5.5) containing Triton X-100 (0.1% v/v) at -20°C for 40 min. The cultures were then centrifuged at 800 × g for 5 min. The supernatant was transferred to new fresh tubes. Approximately 500 μL PBS were added to each tube. TRAP enzyme activity was assayed using p-nitrophenyl phosphate (pNPP) as substrate in an incubation medium containing 10 mM pNPP, 0.1 M sodium acetate (pH 5.8), 0.15 M KCl, 0.1% (v/v) Triton X-100, 10 mM sodium tartrate, 1 mM ascorbic acid and 0.1 mM FeCl_3_. The p-nitrophenol was liberated into p-nitrophenylate after 1 h of incubation at 37°C, and the reaction was stopped by adding 500 μL of 0.6 M NaOH. The absorbance was immediately taken at 405 nm using a spectrophotometer. TRAP activity is expressed as hydrolysis of 1 μM pNPP per minute at 37°C. The specific activity was determined by unit activity per total protein content (mg) as mentioned earlier. The TRAP specific activity was presented in percentage value, which was compared to TRAP specific activity in control cells (cells culture in standard growth medium) that act as basal activity (100%).

### Molecular Assay by RT-PCR

Total RNA was isolated from both types of cells using TRI-Reagent (Sigma, USA) according to the manufacturer's instructions. Absorbance at 260 nm and 280 nm was used to measure the RNA purity; an A260:A280 ratio of 1.8-2.0 was acceptable. Reverse Transcriptase polymerase chain reaction (RT-PCR) was performed with AMV reverse transcriptase and T*fl *DNA polymerase in the Access RT-PCR System (Promega Corporation, USA). The primers were designed by using Primer Premier 5.0 based on the sequences of *Gapdh *(accession number: NM 008084), *Opn *(accession number: X51834) *CatK *(accession number NM 007802) and *Sca-1 *(accession number: NM 007802) obtained from NCBI. Table 1 shows the specific primer sequences that have been designed for RT-PCR reaction.

**Table 1:** Primers involved in RT-PCR reaction.

Genes	Primers	Sequence	Expected Product Size (bp)
*Gapdh*	Forward	^5' ^CACTCCAATCGTCCCTACA ^3'^	
	Reverse	^5' ^AAGGTGGAAGAGTGGGAG ^3'^	717

*Opn*	Forward	^5' ^CACTCCAATCGTCCCTACA ^3'^	
	Reverse	^5' ^GCTGCCCTTTCCGTTGTT ^3'^	234

*CatK*	Forward	^5'^GGCAGGGTCCCAGACTCCAT^3'^	
	Reverse	^ 5'^GTGTTGGTGGTGGGCTAC^3'^	350

*Sca-1*	Forward	^5'^GGCAGGGTCCCAGACTCCAT^3'^	
	Reverse	^ 5'^GTGTTGGTGGTGGGCTAC^3'^	167

*Gapdh; *glyceraldehyde-3-phosphate dehydrogenase as housekeeping gene, *Opn*; Osteopontin as osteoblast marker, *CatK*; Catepsin K as osteoclast marker, *Sca-1*; Stem cell antigen-1 as murine hematopoietic stem cell marker.

The RT-PCR was carried out in a Mastercyler Gradient (Eppendorf, Germany). Reverse transcription (RT) was performed in a program of 45°C for 45 min and pre-denaturation at 94°C for 2 min. The amplification program was as follows: 40 cycles of three-step amplification (denaturation, 94°C for 30 s; annealing, 62°C (*Gapdh *and *Opn*) or 63°C (*CatK *and *Sca-1*) for 60 s; and extension, 68°C for 60 s). A final extension step at 68°C for 7 min was then carried out. Detection of the PCR amplicons was performed by size fractionation by 1% (w/v) agarose gel electrophoresis.

### Von Kossa and May Grunwald-Giemsa Staining

Approximately 5 × 10^5 ^cells/mL were centrifuged at 75 × g for 5 min. The pellet was smeared onto a glass slide and left to air-dry for about 1 to 2 h. The cells were then labeled with von Kossa and May Grunwald-Giemsa staining for osteoblasts and osteoclasts, respectively. ^21^

For von Kossa staining, the cells were fixed with 10% (v/v) formalin in PBS for 30 min and washed with deionized water three times. Then, the cells were stained with freshly prepared 5% (v/v) silver nitrate solution for 30 min and washed well with deionized water three times. Next, the cells were developed with fresh 5% (v/v) sodium carbonate in 25% (v/v) formalin more than 5 min for mineral and matrix staining. After three washes with deionized water, the cells were finally fixed with 5% (v/v) sodium thiosulfate for 2 min to remove un-reacted silver nitrate. Finally, the cells were washed well with deionized water three times and air-dried.

May Grunwald-Giemsa staining was carried out with May-Grunwald's eosinmethylene blue solution (Merck, Germany) and Giemsa solution (Sigma, Germany). May Grunwald-Giemsa staining started with slides immersed in 100% (v/v) May-Grunwald for 4 min. The slides were then transferred directly to 4% (v/v) Giemsa for 4 min and briefly rinsed with distilled water. Excess dye was wiped and air-dried. The von Kossa- and May-Grunwald Giemsa-stained areas were viewed by light microscopy. Approximately 400 cells were counted randomly from each slide. Each experiment consists of 3 different slides. Each generated data was based on 3 independent experiments. Statistical analyses were done using paired t-test using Microsoft Excel 2007 program.

## Competing interests

The authors declare that they have no competing interests.

## Authors' contributions

Contributions: M.D.Y conducted labwork, generated and analyzed data, and wrote the manuscript; R.M.A.W, S.S. and M.A.R analyzed data; and S.H.Z.A designed, analyzed generated data and also involved in writing the manuscript. All authors read and approved the final manuscript.
